# Negative Feedback Enables Fast and Flexible Collective Decision-Making in Ants

**DOI:** 10.1371/journal.pone.0044501

**Published:** 2012-09-12

**Authors:** Christoph Grüter, Roger Schürch, Tomer J. Czaczkes, Keeley Taylor, Thomas Durance, Sam M. Jones, Francis L. W. Ratnieks

**Affiliations:** 1 Laboratory of Apiculture and Social Insects, School of Life Sciences, University of Sussex, Falmer, United Kingdom; 2 Social Evolution Research Group, School of Life Sciences, University of Sussex, Falmer, United Kingdom; Université Paris 13, France

## Abstract

Positive feedback plays a major role in the emergence of many collective animal behaviours. In many ants pheromone trails recruit and direct nestmate foragers to food sources. The strong positive feedback caused by trail pheromones allows fast collective responses but can compromise flexibility. Previous laboratory experiments have shown that when the environment changes, colonies are often unable to reallocate their foragers to a more rewarding food source. Here we show both experimentally, using colonies of *Lasius niger*, and with an agent-based simulation model, that negative feedback caused by crowding at feeding sites allows ant colonies to maintain foraging flexibility even with strong recruitment to food sources. In a constant environment, negative feedback prevents the frequently found bias towards one feeder (symmetry breaking) and leads to equal distribution of foragers. In a changing environment, negative feedback allows a colony to quickly reallocate the majority of its foragers to a superior food patch that becomes available when foraging at an inferior patch is already well underway. The model confirms these experimental findings and shows that the ability of colonies to switch to a superior food source does not require the decay of trail pheromones. Our results help to resolve inconsistencies between collective foraging patterns seen in laboratory studies and observations in the wild, and show that the simultaneous action of negative and positive feedback is important for efficient foraging in mass-recruiting insect colonies.

## Introduction

Positive feedback is the basis for the emergence of many different types of collective behaviours in a wide range of organisms from bacteria to mammals, including group migration, aggregation, nest-site choice, nest construction, and collective foraging [1]–[8]. One consequence of strong positive feedback is that groups may focus on or chose only a sub-set of the available options [4], [6], [9]–[14]. For example, rather than exploiting two identical resources equally, groups of spiders [15], cockroaches [2] or ants [9], [12] often predominantly use just one site. This “symmetry breaking” can be a consequence of the amplification of small, sometimes random, differences in the amount of socially-transmitted information, for example ant trail pheromones, favouring one of the options [12], [16]–[18]. Species of ants and stingless bees with mass-recruitment of foragers via trail pheromone often show limited ability to switch to a better food source because of the strong non-linear response of recruits to the pheromone [4], [9], [10], [13], but see [19]. For example, in a classic experiment Beckers et al. [9] found that *Lasius niger* ant colonies were unable to switch from a low quality feeder to a high quality feeder that became available later. Inflexibility in the reallocation of foragers to newly appearing food sources is surprising because it could lead to reduced colony foraging efficiency, especially in natural environments where changes in food source profitablity and location are inevitable. On the other hand, it has been suggested that focusing on one or a few food sources is advantageous because it helps a colony to defend these against competitors or predators [4], [6], [17].

Information about the dynamics of forager allocation in natural environments in mass-recruiting ants such as *Lasius niger* is scarce. However, one study suggests that while competition does affect the number of foragers at natural food sources, the allocation of ant foragers in nature seems to differ from that observed in laboratory studies [20]. In particular, under natural conditions colonies do seem to be able to allocate foragers according to food source profitability in ways that suggest collective flexibility rather than the strong symmetry breaking and near-irreversible collective decisions seen in laboratory studies [20]. Dreisig [20] found that in several ant species the presence of more workers at natural food sources decreases the rate of energy gain per individual, suggesting that workers inhibit each other’s energy intake. This suggests that reduced individual gains due to crowding may cause negative feedback. Negative feedback such as from crowding can counterbalance positive feedback [6], [7], [17], [18], [21]–[25] and crowding has been shown to lead to an ideal free distribution of cockroaches underneath shelters [2] and more equal traffic flow in foraging *L. niger* ants [26] with access to two parallel pathways on the main trail to the nest.

Although effects of crowding on the foraging behaviour of individual ants have been shown, and the potential for crowding to affect the collective exploitation of food sources has been recognised [17], [27]–[29], the critical experiments have not yet been performed to show that crowding can prevent “symmetry breaking” in foraging ants. In addition, the hypothesis that negative feedback allows flexibility in a changing environment remains untested. Our study addresses these hypotheses in two ways. First, we used laboratory colonies of *L. niger* to investigate the effect of crowding at food sources on forager allocation under both stable and changing food source distributions. Second, we built an agent-based simulation model to test the role of crowding in the same situations. A stable foraging environment was set up by simultaneously offering each colony two identical food sources. The changing environment was set up by initially providing just one food source, with a second, better food source being provided 15 minutes later by which time this first source was already being exploited. In both experiments, in order to vary the strength of any negative feedback due to crowding, we varied the number of feeding holes, thereby mimicking food patches of different sizes. We hypothesized that in the stable situation an increase in the strength of the negative feedback due to crowding would lead to a more even distribution of foragers. In the changing environment we hypothesized that negative feedback caused by crowding at the first food source would allow colonies to reallocate foragers to the second, better, food source.

## Methods

### Study Species

We studied six colonies of *Lasius niger* collected on the University of Sussex campus. Like many ants, *L. niger* collect carbohydrates in the form of honeydew secreted by aphids and nectar from flowers [30]. Experimental colonies were housed in plastic foraging boxes (40×30×20 cm high) containing a circular plaster nest box (15 cm diameter, 2 cm high). The colonies were queenless and had 2400–4700 workers (individually counted at the beginning of the experiments). Queenless colonies forage, make trails and are frequently used in foraging experiments [26], [31]–[33]. Colonies were fed 3 times per week with a mixture of honey, raw egg and agar and given water *ad libitum*. Colonies were deprived of food for 4 days prior to a feeding trial to ensure that the ants were motivated to forage and recruit nestmates to a sucrose syrup feeder. Pheromone deposition in *L. niger* is a very characteristic behaviour and is easily observed. To deposit pheromone on the substrate, a forager interrupts her walk for a fraction of a second and curves the abdomen to touch the substrate with the tip [34]. Only successful foragers deposit pheromone in *L. niger* [34].

### Experimental Setup

Ants were given access to a T-shaped trail system with an 18×2 cm stem and two 10×2 cm branches. The end of each branch widened into a circular platform 8.8 cm in diameter to accommodate a feeder. The entire apparatus was covered in standard printer paper that was replaced after each trial. This was to ensure that the foraging substrate for each trial was unmarked by ant pheromones or other secretions. A 1 M sucrose feeder was placed on each circular platform. The distance between the two feeders was approx. 30 cm (branch + platform). Each feeder consisted of a sealed petri-dish, 5 cm in diameter, with a number (1, 3, 9 or 27) of 1 mm diameter holes in the base (Fig. S1). The ants stood underneath the feeder to collect syrup. The feeder was raised on four 2 cm long disposable wooden legs (Fig. S1). The holes were large enough for up to 8 ants to feed simultaneously at any one hole. Sucrose solution was available in unlimited quantity.

### Experiment 1: Stable Environment with Two Identical Food Sources

We used four different feeder combinations to create different levels of crowding by using two identical feeders each with 1, 3, 9 or 27 holes. Each of the six colonies was tested in each of the four combinations. Each trial lasted for 120 minutes from the time the first ant started feeding. The number of ants feeding and the number of unoccupied feeding holes on each feeder were counted every 5 minutes. The number of full and empty ants leaving the feeder and the number of pheromone depositions on each branch were counted for 2 minutes every 15 minutes. Full ants are easily recognised by an observer by the extended and striped (separated abdominal segments) abdomen. To facilitate counting, a 6 cm section on each branch was marked on the substrate paper and ants and pheromone depositing behaviours were counted on this section. Two observers collected these data, one per 6 cm section. Additionally, the 2 sections were filmed with a high definition video camera (Sony HDR-XR520) to analyse whether empty ants leaving a feeder chose the branch leading to the second feeder or the branch leading to the nest.

### Experiment 2: Changing Environments with Unequal Access to Equal-concentration Food Sources

In this experiment, the second food source was introduced 15 minutes after the discovery of the first food source. The second food source had 3 times as many feeding holes as the first. The feeder combinations were 1 versus 3 holes, 3 versus 9, and 9 versus 27 holes. Each of the six colonies was tested in each of the three combinations. A trial lasted 90 minutes from the time the first ant started feeding. Fifteen minutes later, the second feeder was introduced and was usually discovered within 3 minutes. The number of ants at each feeder and the number of unused feeding holes were counted every 2 minutes for 90 minutes.

### The Agent Based Simulation Model

We developed a spatially explicit agent-based model of foraging agents using NetLogo 4.1.2 [35] (the NetLogo file can be found in the online material (NetLogoFile S1). Please rename the file extension from *.txt to *.nlogo). The model description follows the ODD (Overview, Design concepts, Details) protocol [36], [37].

### Purpose

The purpose of the model was to explore the effects of different crowding thresholds on the allocation of foragers as described in experiments 1 and 2. Additionally, we tested the role of pheromone decay rates on forager allocation in a changing environment. The model is not intended to be an exact and fully parameterized model of *L. niger* foraging. While the modelled situation is based on our experimental set-up, the aim was to build a more generic model that captures the key elements of ant foraging and recruitment to investigate how crowding affects worker allocation in a species with strong positive feedback via pheromone trails and negative feedback via crowding at food sources.

### Model entities, State Variables and Scales

For most simulations, we used 500 agents (see Table 1 for parameters), which corresponds approximately to the number of ants that can be expected to forage during a typical experimental trial using colonies with several thousand ants (i.e., not all the ants in a colony forage). Agents could assume any one of 6 different states: idle inside the nest, searching for food, feeding at a food patch, at a food patch but unable to feed due to crowding (dissatisfied), laying a pheromone trail while returning to the nest (recruiter), unloading.

**Table 1:** Overview of processes, parameters and default values used in the model.

Colony size	500 agents
Leaving rate of idle foragers	(2/1000 * no. agents in the nest)/sec
Crowding threshold	8, 24, 72, 216
Drinking time	60 time steps (60 seconds)
Return-to-nest time	ca. 40 time steps
Unloading time	60 time steps
Time delay between introduction of both food sources	0 time steps (Part C); 900 time steps (Part D)
Amount (*c*) of chemical deposited per patch	60 pheromone units
Pheromone decay rate r	0.4 (corresponds to a decay in c. 2700 time steps or 45 min)
Amount of pheromone at t	C_i_(t) = C_i_(t–1)×(100−r)/100
Pheromone detection threshold per patch	0.05 pheromone units

The simulated agents occupied a specific location at every point in time and were located on a two-dimensional square grid with the shape of a T-maze connected to the nest. The default branch width was 4 squares, the stem width was 5 squares. The default lengths were 24 squares for the stem and 11 for each arm. The nest was located at the base of the T-maze (5×4 squares), with one food patch at the end of each branch (4×4 squares). Multiple agents could occupy the same square. Simulations were run in discrete time steps (*t*). One time step was made to correspond approximately to one second in the experiment in the following way. It took real ants approximately 40 seconds to walk from a food source to the nest. Hence, for the model we chose branch and stem lengths that required approximately 40 time steps with an agent walking-speed of 1 square per time step (agents did not always walk in a perfectly direct way from nest to feeder). Thus one time step in the model corresponds to one second. Total model running time was 5400 time steps, corresponding to approximately 90 minutes. Because of the stochastic nature of the model, 30 model runs were performed for each combination of parameter values. For each run, the random number generator was uniquely seeded based on the operating system’s time and date [35].

### Model Process Overview and Scheduling

Agents that left the nest started to perform a random walk (searching) until a food patch or, at a later stage of the simulation, a pheromone trail was encountered. Agents finding a food patch spent 60 time steps taking on food at the patch if there was no crowding. Successful agents then walked directly to the nest and then took 60 time steps to unload. After unloading, agents could leave the nest and start searching again (random walk) or follow a trail. If food patches were crowded, agents became dissatisfied.

During the simulation, the behavioural states and variables were updated for each agent at every time step. The different states were updated asynchronously in sequence (idle agents→foraging agents→feeding agents→dissatisfied agents→recruiting agents→unloading agents). However, the model was robust to changes in the sequence (see Fig. S2).

In model 1 (corresponding to experiment 1), two identical food sources were offered simultaneously. We found that 1 feeding hole can accommodate a maximum of 8 foragers. Hence, we again used 4 different crowding thresholds, which corresponded to the crowding levels in the experiment: high (8 agents ≈ 1 feeding hole), medium (24 agents ≈ 3 feeding holes), low (72 agents ≈ 9 feeding holes) and very low (216 agents ≈ 27 feeding holes). For simplicity, crowding was modelled as an all-or-nothing state. For example, if 8 agents were already present at a food patch in the high crowding situation, other agents at the feeder location could no longer access the food and became dissatisfied. Apart from crowding, food patches were *ad libitum* as in the experiments.

In model 2 (corresponding to experiment 2), one food source was introduced with a delay of 900 time steps (∼15 minutes). This second food source permitted 3 times as many agents access to forage before the crowding threshold was reached (8 vs. 24 agents, 24 vs. 72 agents, 72 vs. 216 agents). If the number of agents on a food patch was higher than the crowding threshold for this patch, a newly arrived agent became dissatisfied and performed a random walk.

### Model Design Concepts

The pheromone deposited on the trail system and the proportions of agents at the two food sources, as influenced by both negative and positive feedback, are emergent properties of the model. The concepts of adaptation, objectives and prediction are not important in this model. There is no learning in the model.

Sensing is important in this model: agents leaving the nest were able to detect pheromone left on patches and oriented themselves according to the amount of pheromone. Agents that had fed successfully at either food source, and were walking back to the nest were assumed to know the direction of the nest (implemented by means of a nest odour). Furthermore, agents reaching the food patch were able to perceive whether the number of agents on a food patch equalled the crowding threshold for this patch (implemented by counting the number of feeding agents on the patch.

Stochasticity is used to introduce variability in the number of agents leaving the nest at any time step (Table 1) and in their random walks.

### Model Initialisation

At the beginning of each simulation trial, the nest, the T-maze and the food sources were initialised as described above. The amount of pheromone chemical was set *C_pheromone_* = 0 for all patches, and the nest scent of patch *X* was set to *C_nest_* = 100−distance (*X_nest_*, *X*), where *X_nest_* is the patch at the centre of the nest. All agents were initiated at the nest centre and their state set to idle, with a probability *P_leave_* (Table 1) to leave the nest.

### Submodels

The move-foraging submodel defined how agents behave after leaving the nest: Agents could follow a pheromone trail by sampling 3 patches in walking direction (0°, 45° left and 45° right) and walk towards the direction of the patch with most pheromone. Pheromone was detected if the amount exceeded a threshold level of pheromone (Table 1). We assumed the pheromone chemical to be volatile, and chose an evaporation rate that led to a decay of the pheromone trail below the perception threshold of the agents that was equivalent to approximately 45 minutes. This was based on the pheromone strengths we measured during test runs and corresponds to experimentally measured values for *L. niger* [38]. The decay of pheromone was calculated for each square of the grid at each time step as *C_i,t_ = C_i,(t –1)_×(100−r)/100*; where *C_i_* is the chemical on patch *i, r* is the evaporation rate in % and *t* is the time point, leading to an exponential decay (see also Table 1). If the 3 patches in walking direction had no pheromone or below-threshold pheromone levels, then the agent moved in a random direction (towards 8 possible patches).

The move-to-nest submodel defined the behaviour of agents after successful foraging. Full agents perceived the strength of the nest-odour (see section Initialisation) on patches and behaved as they did in the case of pheromone. In nature, ants find their nest relying on various methods such as land-mark learning, path-integration and olfaction [39]–[41]. For the purpose of this model, the method of finding the way back to the nest was irrelevant. The quality of food patches was high in that all successful agents deposited a pheromone trail with amount *c* on each patch they cross when walking back to the nest. This is a simplification as in nature not all ants deposit trail pheromones and ants also deposit pheromone when walking from the nest to the food source [42]. However, for the purpose of our model this was irrelevant, because we simply wanted the agents to establish an attractive pheromone trail with a certain decay rate.

Dissatisfied agents performed a random walk without paying attention to the pheromone trail or laying a pheromone trail. For all agent movements described above, agent step size was equivalent to 1 patch length independent of direction. Movements were therefore off-lattice.

### Sensitivity Analysis

In order to test how strongly our results depended on the values of key parameters we systematically varied the number of agents, pheromone decay rates, side branch lengths, and the length of the stem. The model was robust over a wide range of these parameters. Some deviations (e.g. with larger T mazes) are given in the Results section of the paper.

### Statistical Analysis

We used linear mixed-effect models (LME) and the statistical package R 2.9 [43] to analyse the experimental data. R fitted the models with the lme-function of the nlme-package [44]. In experiment 1, the response variables in the different models were (i) the relative difference between the two branches, (ii) the number of empty ants leaving the nest and (iii) the ratio between empty and full ants leaving the nest. In experiment 2, the response variable was the proportion of ants foraging at the second feeder. We included colony and trial as hierarchically nested random effects to control for the non-independence of data points from the same colony and the same trial [44]. If necessary, we transformed the response variable with a square-root transformation to achieve a normal distribution. For model selection we used the protocol proposed by [44]. We first explored the optimal structure of the random components (comparing random intercept models with random intercept and slope models). We then explored the significance of the fixed effects. Our fixed effects were the number of holes and time of measurement. Time of measurement was included because previous studies showed temporal changes in forager allocation in similar experiments, e.g. [9]. The interaction between the two fixed-effects was removed for the final model if it was not significant (p>0.05). The final model always included both fixed-effects. If we tested datasets multiple times, we adjusted the significance levels using the sequential Bonferroni method [45].

## Results

### Experiment 1: Stable Environment with Two Identical Food Sources

**Figure 1:**
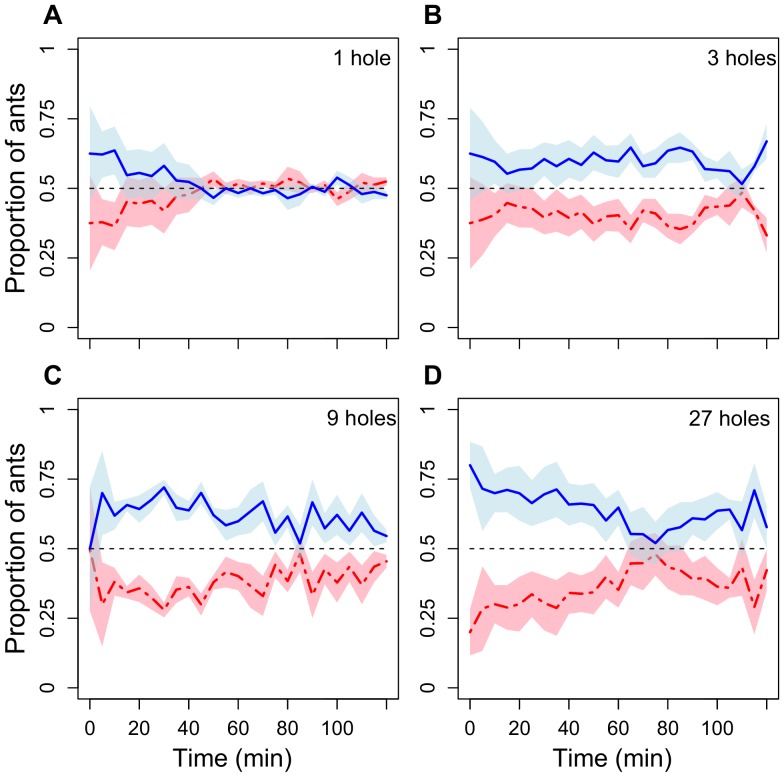
Proportions of ants visiting two identical 1 molar sucrose feeders each with 1, 3, 9 or 27 feeding holes (Experiment 1). The blue line represents the feeder that had more ants after 5 minutes, the red line the other feeder. The dashed black line indicates an equal distribution of ants at both feeders. Data represent the mean of 6 test colonies, 1 trial per colony for each number of holes. The shaded areas (light blue and pink) represent the standard errors (SE) of the mean.

**Figure 2:**
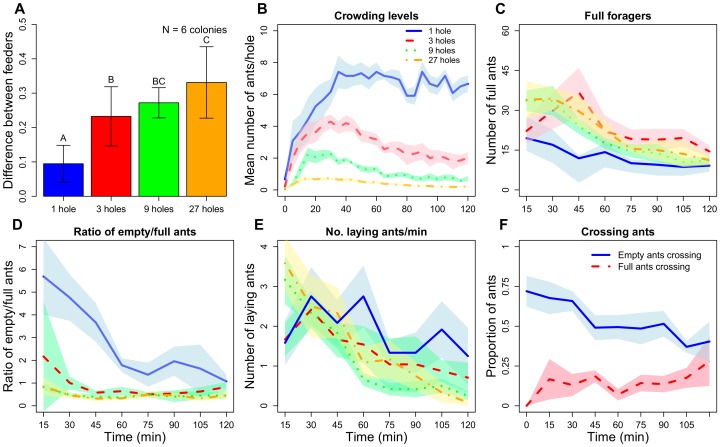
The number and behaviour of ants on both branches (Experiment 1). (A) Mean average difference in the proportions of ants feeding at the two feeders during the whole of the 120 minute trial. Bars show the mean and standard error for the 6 test colonies, one trial each per treatment. The letters above the bars indicate statistically significant differences (linear mixed-effect models: P<0.05; see results for details). (B) Mean number of ants per hole during the whole of the trial. (C) The mean number of successful, i.e. full ants leaving a feeder (averaged for the two feeders) counted over 2 minutes. (D) The mean ratio of empty to full ants returning to the nest for the 6 colonies, measured every 15 min. (E) The mean number of ants laying trail pheromone on either branch. Ants were counted during 2 min every 15 min. (F) The mean proportion of empty (blue line) and full (red line) ants leaving a feeder under high crowding conditions (both feeders had 1 hole) and walking towards the other feeder instead of back to the nest. As can be seen the proportion of empty ants walking towards the other feeder was considerable higher than the proportion of full ants. The shaded areas represent the SE of the mean.

When a trial began, the feeders were discovered within a few minutes and a rapid build up of foragers was observed. When both feeders had 1 feeding hole (1∶1) both had very similar numbers of ants (Fig. 1A). Conversely, when both had 9 (9∶9) or 27 (27∶27) holes, foraging activity was strongly biased towards one feeder (Fig. 1C,D). An intermediate pattern is found when both feeders had 3 (3∶3) holes (Fig. 1B). When feeders had 9 (9∶9) or 27 (27∶27) holes, the feeder that had more foragers after 5 minutes was usually (11 of 12 trials, which is significantly different from the 50∶50 random expectation: χ^2^ = 8.33, df = 1, p = 0.004) the feeder that was exploited more, on average, during the entire 120 minute trial. The relative difference in the number of ants foraging at the two feeders differed significantly between treatments (LME, random intercept and random slope [for “time”] model: t-value = 4.58, p = 0.0003; Intraclass Correlation Coefficient (ICC): colony = 0.45; trial = 0.12; Fig. 1 and Fig. 2A; see Table 2 for pair-wise comparisons). Overall, the differences between the two feeders tended to decreased over time (t-value = −1.86, p = 0.064). When analysing each treatment separately, we found that the proportion of ants feeding at the feeder that had more ants after 5 min was not different from 0.5 when both feeders had 1 hole (0.51±0.04 [mean±SD], one-sample t-test: t-value = 1.37, df = 22, p = 0.18). If feeders had more holes, the proportion of ants feeding at this feeder was significantly higher than 0.5 (3∶3 holes, 0.59±0.04, t-value = 12.7, df = 22, p<0.0001; 9∶9 holes, 0.62±0.05, t-value = 11.3, df = 22, p<0.0001; 27∶27 holes, 0.63±0.06, t-value = 10.6, df = 22, p<0.0001.

**Table 2:** Effects of the number of feeding holes on the difference in the proportions of ants visiting two identical (Exp 1) or two different (Exp 2) feeders, the ratio between ants returning full or empty from the feeder.

Exp 1 - Difference between identical feeders	t-value	p-value
1 vs. 3 holes	2.95	<0.0099*
1 vs. 9 holes	4.96	<0.0002*
1 vs. 27 holes	6.34	<0.0001*
3 vs. 9 holes	2.01	0.063
3 vs. 27 holes	3.39	<0.004*
9 vs. 27 holes	1.39	0.19
Empty ants leaving feeder		
1 vs. 3 holes	−2.56	0.01*
1 vs. 9 holes	−4.38	<0.0001*
1 vs. 27 holes	−4.72	<0.0001*
3 vs. 9 holes	−1.81	0.071
3 vs. 27 holes	−2.16	0.031
9 vs. 27 holes	−0.35	0.73
Effect of time	−9.85	<0.0001
Ratio empty/full ants		
1 vs. 3 holes	−6.6	<0.0001*
1 vs. 9 holes	−9.2	<0.0001*
1 vs. 27 holes	−10.83	<0.0001*
3 vs. 9 holes	−2.66	0.018*
3 vs. 27 holes	−4.28	<0.0007*
9 vs. 27 holes	−1.61	0.13
Effect of time	−4.97	<0.0001
Exp 2– difference between non-identical feeders		
1/3 holes vs. 3/9 holes	−2.06	<0.066
1/3 holes vs. 9/27 holes	−4.38	0.0014*
3/9 holes vs. 9/27 holes	−2.32	<0.043

* Significant after sequential Bonferroni correction. The raw data for the tests presented in this table is provided in the supplementary material (Table S1).

Figure 2b shows that crowding, quantified as the number of syrup-drinking ants per feeding hole, was negatively correlated with the number of holes. The fact that feeder use was also more similar at 3 (3∶3) holes versus 27 (27∶27) holes (Fig. 2A) shows that also moderate levels of crowding (Fig. 2B) cause enough negative feedback to have some balancing effect. The number of feeding holes affected the number of unsuccessful (empty) ants leaving a food source (first 60 min of experiment: LME, random intercept model: t-value = −3.16, p = 0.0056; ICC: colony = 0.21; trial = 0.50). More empty ants left the feeder if it had only 1 feeding hole (pair-wise comparisons shown in Table 2). The number of full ants leaving a feeder is shown in Fig. 2C. As a consequence, the resulting ratio between empty and full ants leaving the feeders was also affected by the number of feeding holes (Fig. 2D). All ratios differed significantly between treatments except 9 versus 27 holes (Table 2). Our videos also showed that a substantial proportion of empty ants leaving the feeder under high crowding conditions (1∶1) walked towards the second feeder, instead of walking towards the nest (Fig. 2F). The probability of full ants to walk to the second feeder instead of returning to the nest was much lower (Fig. 2F, LME, random intercept model: t-value = −6.8, p<0.0001; ICC for colony = 0.12). Overall, the proportion of ants walking back to the nest increased with time (t-value = −3.1, p = 0.003), probably due to satiation (Fig. 2F). The same model also showed an interaction between “time” and “state” (full or empty) (t-value = 2.91, p = 0.005), indicating that the difference between full and empty ants in their propensity to walk back to the nest decreased over time.

### Experiment 2: Changing Environments with Unequal Access to Equal-concentration Food Sources

**Figure 3:**
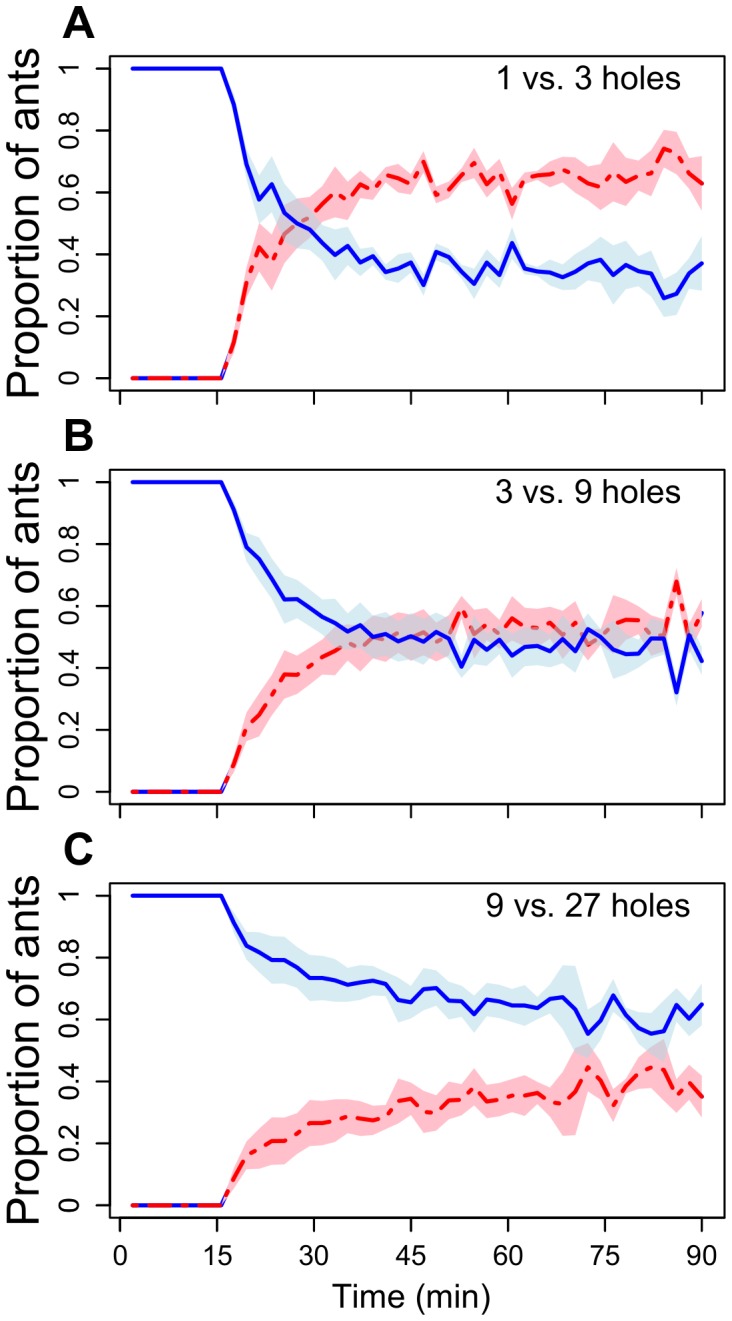
The mean proportion of ants at the two feeders, in which the second feeder (red line) had three times as many feeding holes but was made available 15 minutes after ants starting collecting syrup at the first feeder (blue line) (Experiment 2). As can be seen, the lines cross for the 1 versus 3 hole situation, but not for 9 versus 27. The shaded areas represent the SE of the mean.

This experiment investigated how crowding affects the allocation of foragers to a second feeder made available 15 minutes after the first, but with 3 times as many feeding holes. Colonies with high levels of crowding at the first feeder (1∶3) quickly, within an average of 10 minutes, reallocated the majority of foragers to the new 3-hole feeder (seen by the crossing of lines in Fig. 3A). Conversely, colonies continued to allocate foragers mainly to the first feeder when the first feeder had generous feeding access, 9 holes (9∶27; Fig. 3C). A situation with moderate crowding (3∶9) leads to an intermediate pattern. The proportion of foragers visiting the second feeder during the last 50 minutes of a trial differed significantly between the treatments (LME, random intercept model: number of holes: t-value = −4.12, p<0.00171; effect of time: t-value = 3.38, p = 0.0008; ICC: colony<0.001; trial = 0.44; pair-wise comparisons are shown in Table 2). The significant effect of time shows that, overall, the proportion of ants feeding at the second feeder increased during the last 50 minutes of the experiment.

### Agent-based Models 1 and 2

**Figure 4:**
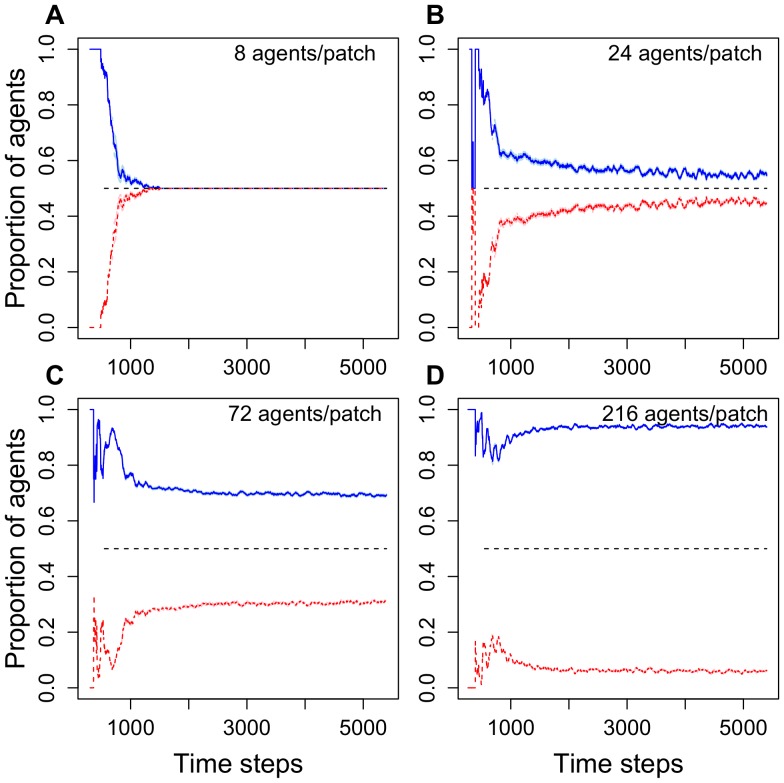
Proportions of agents visiting two identical food patches each with space for 8, 24, 72 or 216 foraging agents (Model 1). The blue line represents the patch that had more agents after 600 time steps, the red line the other. The dashed black line indicates an equal distribution of agents at both feeders. Data averaged from 30 simulations in each situation. The standard deviation (StDev) is shown in light blue and pink. The StDev used instead of the SE because the SE is too small to be seen by eye.

Figure 4 shows how crowding affects the proportion of agents exploiting the food patch that had more agents after 600 time steps (corresponding to 10 minutes). There is a clear effect of the number of agents that can simultaneously forage at a patch on the degree of symmetry breaking. While strong crowding, in which a low number of agents can simultaneously forage at a given patch, leads to a more equal distribution of agents at both food patches (Fig. 4A), low crowding leads to strong symmetry breaking.

**Figure 5:**
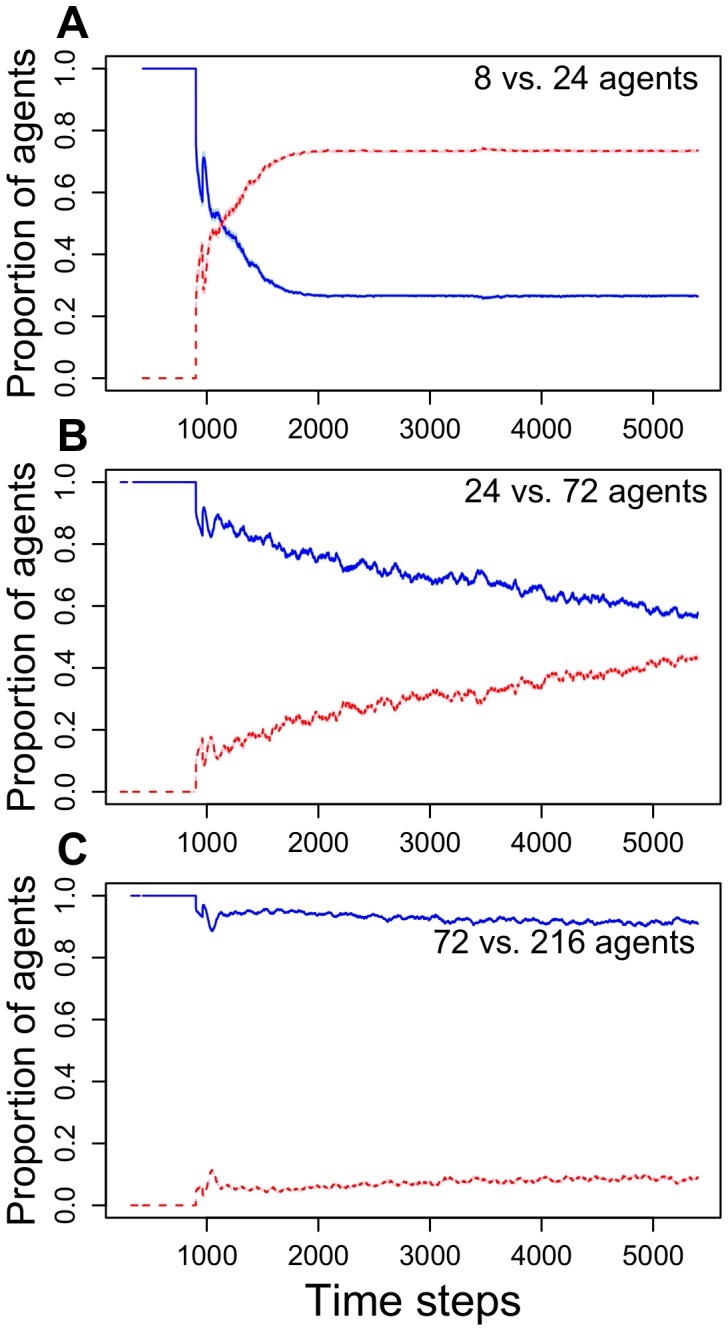
Proportions of agents foraging at the two food patches, in which the second patch (red line) allowed three times as many agents to feed simultaneously but was made available 900 times steps after agents started foraging at the first food patch (blue line) (Model 2). Data averaged from 30 simulations in each situation. The StDev is shown in light blue and pink.

**Figure 6:**
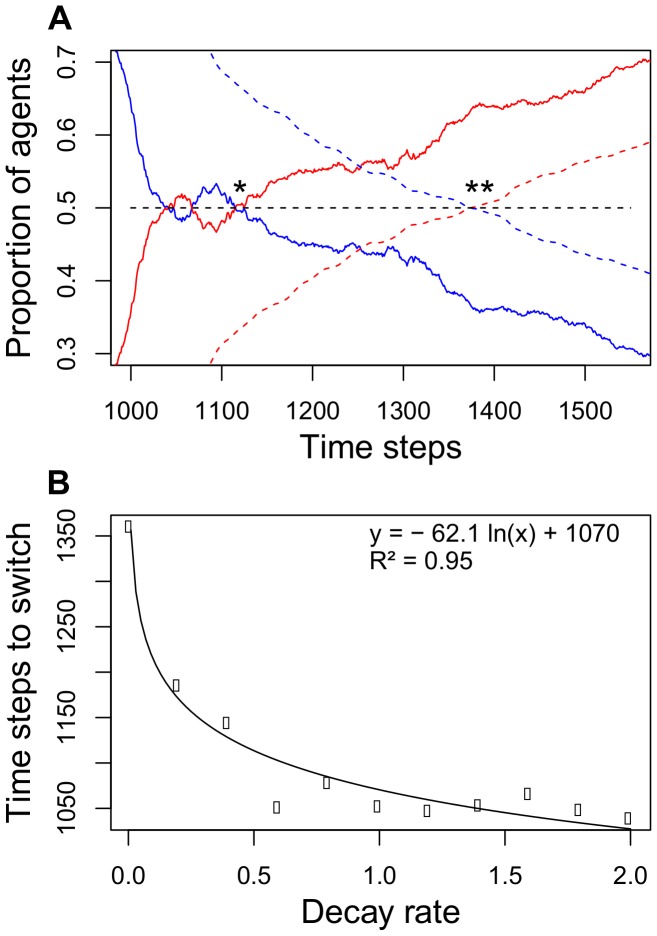
The number of agents on both branches and the time colonies needed to switch. (A) Switch point of agents (solid lines, *) and pheromone trail strength (dashed lines, **). The solid lines show the proportion of ants foraging at the first (blue) and second patch (red). The second patch was available after a 900 time step (15 minutes) delay, but allowed 3 times more agents to collect food simultaneously (8 vs. 24 agents). The dashed lines show the relative amounts of pheromone on the branches leading to the first (blue) and second patch (red). The pheromone switch happened some time after the switch in the number of foraging ants (average of 30 simulations). (B) Relationship between the pheromone decay rate and the time until more agents foraged at the second food patch. Note that the switch happens even with a pheromone decay rate of zero. A decay rate of 2.0 corresponds to a pheromone decay below the perception threshold of the agents in less than 10 minutes.

As in experiment 2, if a superior food patch is made available after a delay, high levels of crowding lead to rapid reallocation of foragers to the superior new patch (Fig. 5A), but without crowding agents do not reallocate (Fig. 5C). Intermediate levels of crowding lead to an intermediate pattern (Fig. 5B). As the switch to the superior patch is more rapid under high crowding conditions this suggests that flexibility does not require pheromone decay. Indeed, the food patch that is introduced with a time delay received more foragers even before its branch had more trail pheromone (Fig. 6A). On average, more agents were present at the second feeder after 1115 time steps, while the amount of pheromone present was only greater after 1374 time steps (averages of 30 simulations). However, pheromone decay rate does affect the time taken to switch: the faster the decay, the faster colonies reallocate agents to the second food patch (linear regression with log[decay rate]: t-value: −13.26, p<0.001, R^2^ = 0.95, Fig. 6B). However, even with zero pheromone decay on the branch leading to the first food patch, colonies can still switch to the more profitable second patch (Fig. 6B).

### Sensitivity Analysis

We tested whether the results of our model are sensitive to changes in the values of some key parameters to determine the robustness of our main findings. We found that colonies as small as 150 foraging agents were able switch to the second food patch at the strongest crowding threshold (8 vs. 24 agents that were allowed to forage simultaneously at a patch, corresponding to 1 vs. 3 feeding holes). Below this colony size, allowing 8 agents to forage simultaneously at the first patch no longer leads to a sufficient number of agents becoming dissatisfied due to crowding to cause a switch (Fig. S3). On the other hand, with the lowest crowding conditions (72 vs. 216 agents/patch, corresponding to 9 vs. 27 feeding holes) only colonies with more than c. 2000 foraging agents switched to the second food source (Fig. S4). The effect of decay rate was relatively small as shown in Fig. 6B. Increasing the length of the stem of the trail system to a distance corresponding to approximately 2 meters did not affect the ability to quickly switch to the second food patch with high crowding conditions (8 vs. 24 agents/patch) (Fig. S5). However, increasing the distance between the food patches, equivalent to longer arm length in a T-maze, had a stronger effect on the time it took for the switch to take place (Fig. S6). At a distance corresponding to about 2 meters between the two food patches, colonies with 500 agents no longer switched (8 vs. 24 agents; Fig. S6C). We also tested if the probability of dissatisfied agents to walk to the nest instead of to the second food source affects how long it takes to switch to the second food source. The simulations show that the time to switch increases if the probability to walk to the nest increases (Fig. S7).

## Discussion

Our results show that crowding results in negative feedback and enables colonies to allocate foragers more evenly between two feeders in a stable environment and to reallocate more foragers to a superior feeder in a changing environment. Our agent based simulation model confirms the role of crowding as a mechanism enabling this group-level flexibility.

Our experimental results indicate that the ability of a colony to switch to a superior food source is unlikely to depend strongly on pheromone trail decay to the first food source, which has been suggested as a potential mechanism [20]. Colonies switched on average after only 10 minutes, which is faster than expected if it were due to pheromone decay given that trail pheromones in *L. niger* persist for at least 40–60 minutes [31], [39]. In addition, even under high crowding conditions ants continued to deposit pheromone when walking to and from a feeder (Fig. 2E). Our simulation supports this conclusion as colonies switched to a superior food source even if the pheromone trail to the first food source had not decayed. Indeed, there is a period during which the branch leading to the first food patch still has more pheromone even though the majority of agents are already foraging at the second food patch. Hence, the relative strength of the pheromone trails on both branches under crowded conditions is a consequence rather than a cause of the switch to the second food source. However, the model also shows that colonies can reallocate foragers more quickly if the pheromone decays faster (Fig. 6B). In our experiments, the two feeders were identical in terms of the sucrose concentration (1 M), which is another important determinant of food source quality. We anticipate that the switch would have happened even faster if the second feeder would have had a higher sucrose concentration, as was the case in previous studies, e.g. [9]. This is because higher sucrose concentration increases the intensity of pheromone depositions [39].

Recent theoretical work suggests that stochasticity in the decision-making process or the use of two different types of pheromones could potentially lead to flexibility in collective decision-making in the ant *Pheidole magacephala* ([19], [46], see also [47]). However, the underlying mechanisms for flexibility in *P. megacephala* require further investigation. Our results demonstrate a simple mechanism in addition to stochasticity in *L. niger*. In crowded situations, many ants are unable to gain sufficient access to the food source, resulting in reduced food source profitability as experienced by individuals. As a consequence, many unsuccessful foragers leave the feeding site (Fig. 2D). A large proportion of these ants did not return to the nest but found the branch leading to the alternative feeder (Fig. 2F), thereby increasing the probability of using this feeder. Overall, unsuccessful foragers were approx. 3.2 times more likely to walk towards the alternative feeder than successful ants (Fig. 2F). The importance of this simple mechanism is supported by the results of the agent models. Here, dissatisfied agents (agents unable to collect food due to crowding) did not deliberately leave the crowded food source to search for another food source but found it via a random walk.

We suggest that in nature these three mechanisms (pheromone decay, stochasticity and searching by unsuccessful foragers) could potentially all result in colony-level flexibility, but would act on different time scales and might be more or less important depending on factors such as the geometry of the trail network and the distances between the food sources. For example, in the simulation model the probability that dissatisfied agents will discover the second food source, and, therefore, the ability of colonies to reallocate foragers, depended on the distance between the two sources (sensitivity analysis). If the two food sources are far apart, dissatisfied agents performing a random walk are less likely to find the second source. On the other hand, the distance of the two food sources from the nest did not affect the ability of colonies to reallocate foragers quickly. Also the angle of the bifurcations have the potential to affect collective flexibility because bifurcation angles have been shown to affect branch choice and the U-turn probability of foragers of other ant species [48]–[50]. We simulated this by varying the probability of ants to walk back to the nest vs. to the second feeder (Fig. S7) and found that this probability indeed affects the speed of switching to the second food source. Depending on species, other feedback signals may also be used, such as pheromonal stop-signals deposited on unprofitable branches in Pharaoh’s ants [21], [22].

Our results help unify understanding of the distribution of an ant colony’s foragers under both laboratory conditions with unrestricted access to food [9] and natural conditions with more restricted availability [20]. In nature, foragers of many ant species depend heavily on honeydew produced by aphids or other Homoptera for their carbohydrate supply [20], [30], [51]. The amount of aphid honeydew produced per patch depends on species and number [20], [51]. The key determinant of aphid patch profitability seems to be the accessible amount rather than the quality of the produced honeydew [51] and ants have been shown to distribute themselves among various patches according to the amount of honeydew produced by each aphid patch [20]. Hence, as in our experiment with high crowding, forager allocation among aphid patches depends on patch profitability rather than the sequence of food patch discovery. This ability to allocate foragers dynamically according to the profitability of food sources is also found in the honey bee, *Apis mellifera*. As in ants, successful honey bee foragers recruit nestmates to profitable food sources, but unlike ants they use the waggle dance [52], [53]. The waggle dance is also a positive feedback mechanism, but the relationship between signal and response is more linear than is the case in ant trail pheromones (see Fig. 5.28 in [53]). As a consequence, honeybee colonies can exploit two identical food sources without symmetry breaking and are able to allocate more foragers to a superior food source that appears later without crowding [17], [53].

In summary, our results show that when strong and non-linear positive feedback occurs, negative feedback can prevent ant colonies becoming trapped in suboptimal collective states. This mirrors the balancing effects of negative feedbacks in other complex systems. In engineering, James Watt’s steam regulator is a classic example and in human physiology a failure in negative feedback in the regulation of blood sugar level causes diabetes. We predict that negative feedbacks will be found to occur widely in other complex biological systems that have strong positive feedback mechanisms, to prevent the system becoming trapped in suboptimal states.

## Supporting Information

Figure S1 **Photo showing the feeder (petri-dish, 5 cm diameter) standing on 2 cm wooden legs.** The feeder contained 1 M sucrose solution. Ants could gain access to the solution via 1 mm feeding holes (27 in this situation).
(TIF)

Figure S2 **Model 1 with the different behavioural states being updated in reversed sequence (unloading agents→recruiting agents→dissatisfied agents→feeding agents→foraging agents→idle agents).** Proportions of agents visiting two identical food patches each with space for 8, 24, 72 or 216 foraging agents. The blue line represents the patch that had more agents after 600 time steps, the red line the other. The dashed black line indicates an equal distribution of agents at both feeders. Data averaged from 30 simulations in each situation. The standard deviation (StDev) is shown in light blue and pink. However, since the StDev is very small it is difficult to see by eye.
(TIF)

Figure S3 **Smallest colony size still showing flexibility under high crowding conditions (8 vs. 24 agents).** Proportions of agents foraging at the two food patches, in which the second patch (red line) allowed three times as many agents to feed simultaneously but was made available 900 times steps after agents started foraging at the first food patch (blue line). Data averaged from 10 simulations in each situation. The StDev is shown in light blue and pink. However, since the StDev is very small it is difficult to see by eye.
(TIF)

Figure S4 **Colony size needed for flexibility under low crowding conditions (72 vs. 216 agents).** Proportions of agents foraging at the two food patches, in which the second patch (red line) allowed three times as many agents to feed simultaneously but was made available 900 times steps after agents started foraging at the first food patch (blue line). Data averaged from 10 simulations in each situation. The StDev is shown in light blue and pink. However, since the StDev is very small it is difficult to see by eye.
(TIF)

Figure S5 **The effect of the main branch length on flexibility under high crowding conditions (8 vs. 24 agents).** Proportions of agents foraging at the two food patches, in which the second patch (red line) allowed three times as many agents to feed simultaneously but was made available 14000 time steps after agents started foraging at the first food patch (blue line). The delay of 14000 time steps was chosen because it guaranteed that agents discovered the first food source by random walks even if the main branch was 10 times longer than by default. A main branch length×10 corresponds to approximately 2 m. The instantaneous switch shown in (A) is caused by a large number of dissatisfied agents occupying the second food patch after 14000 time steps. However, with a longer main branch the dissatisfied agents are distributed over a larger area. Data averaged from 10 simulations in each situation. The StDev is shown in light blue and pink.
(TIF)

Figure S6 **The effect of the arm length on flexibility under high crowding conditions (8 vs. 24 agents).** Proportions of agents foraging at the two food patches, in which the second patch (red line) allowed three times as many agents to feed simultaneously but was made available 1800 time steps after agents started foraging at the first food patch (blue line). The delay of 1800 time steps was chosen because it guaranteed that agents discovered the first food source by random walks even if the arm length was 6 times longer than by default. An arm length×6 corresponds to approximately 2 m. Data averaged from 10 simulations in each situation. The StDev is shown in light blue and pink.
(TIF)

Figure S7 **The effect of the probability of dissatisfied agents to walk to the nest versus to the second feeder on collective flexibility under high crowding conditions (8 vs. 24 agents).** Proportions of agents foraging at the two food patches, in which the second patch (red line) allowed three times as many agents to feed simultaneously but was made available 900 time steps after agents started foraging at the first food patch (blue line). This model slightly differed from the main model in that dissatisfied ants did not perform a random walk but had a certain probability to either walk on a direct path to the nest or to the second food source (probabilities were 10% vs. 90%, 50% vs. 50, 90% vs. 10%). If both feeders were crowded, dissatisfied agents walked back to the nest and then became “foraging agents” again. Data averaged from 10 simulations in each situation. The StDev is shown in light blue and pink.
(TIF)

Table S1 **The raw data for the tests presented in** **Table 2** **.**
(XLSX)

NetLogoFile S1 **The NetLogo computer code of the agent-based simulation model.** The file extension “.txt” can be renamed to “.nlogo” to open the file.
(TXT)
