# Supplementary figures and images for: Negative Feedback Enables Fast and Flexible Collective Decision-Making in Ants
Source: PLoS One. 2012 Sep 12;7(9):e44501. doi: 10.1371/journal.pone.0044501 (PMC3440389; doi:10.1371/journal.pone.0044501)

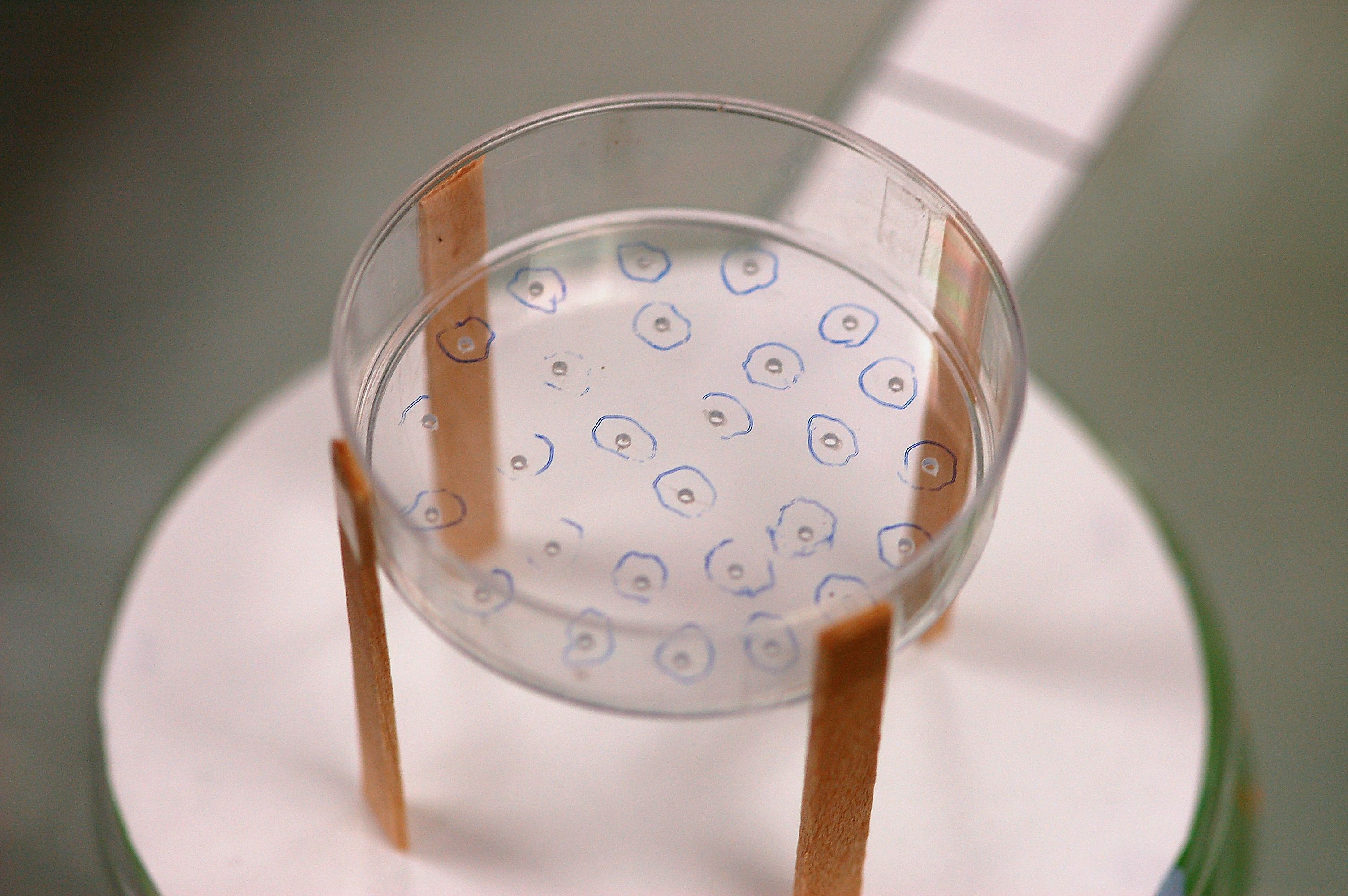

Supplement: Figure S1 — Photo showing the feeder (petri-dish, 5 cm diameter) standing on 2 cm wooden legs. The feeder contained 1 M sucrose solution. Ants could gain access to the solution via 1 mm feeding holes (27 in this situation). (TIF) [file pone.0044501.s001.tif]

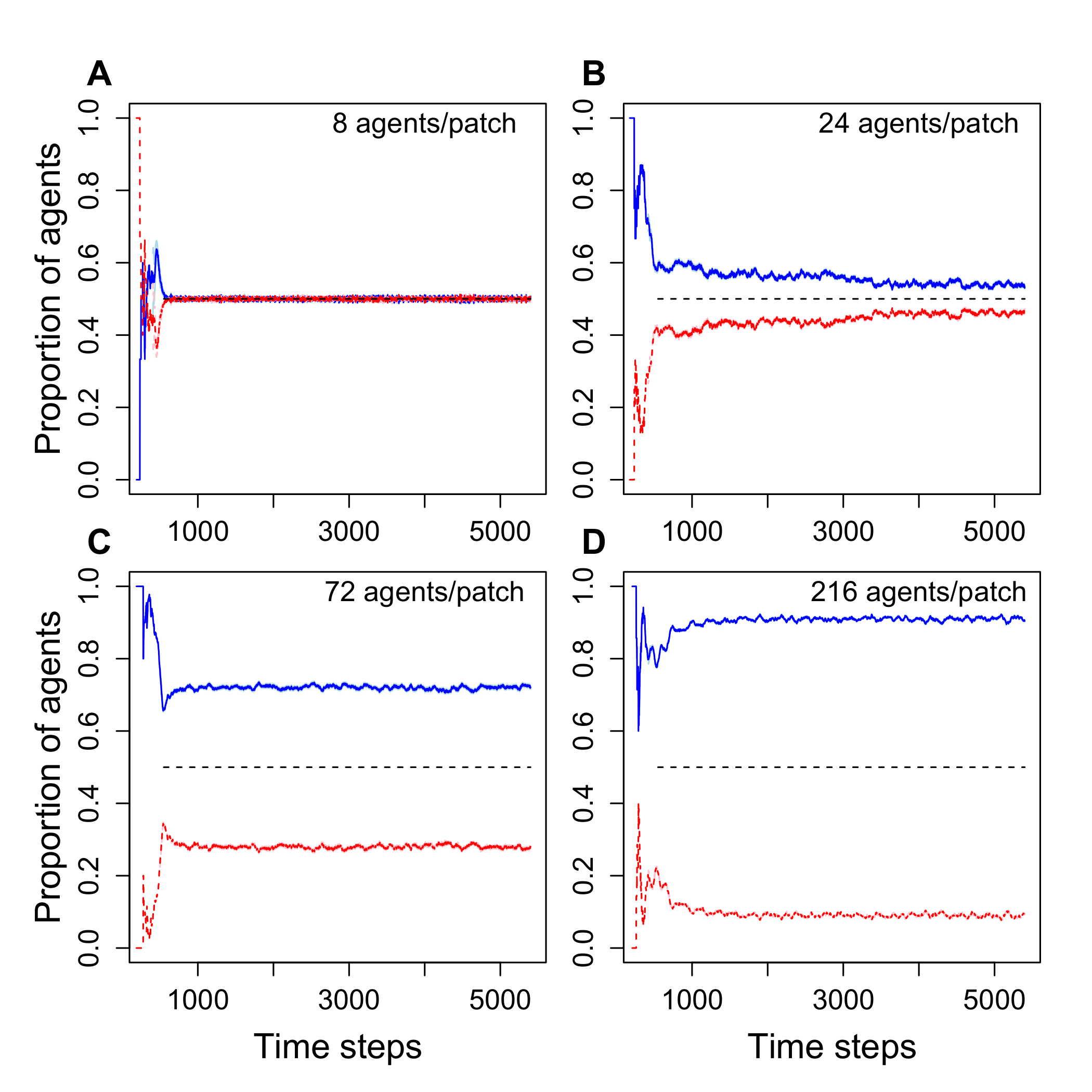

Supplement: Figure S2 — Model 1 with the different behavioural states being updated in reversed sequence (unloading agents→recruiting agents→dissatisfied agents→feeding agents→foraging agents→idle agents). Proportions of agents visiting two identical food patches each with space for 8, 24, 72 or 216 foraging agents. The blue line represents the patch that had more agents after 600 time steps, the red line the other. The dashed black line indicates an equal distribution of agents at both feeders. Data averaged from 30 simulations in each situation. The standard deviation (StDev) is shown in light blue and pink. However, since the StDev is very small it is difficult to see by eye. (TIF) [file pone.0044501.s002.tif]

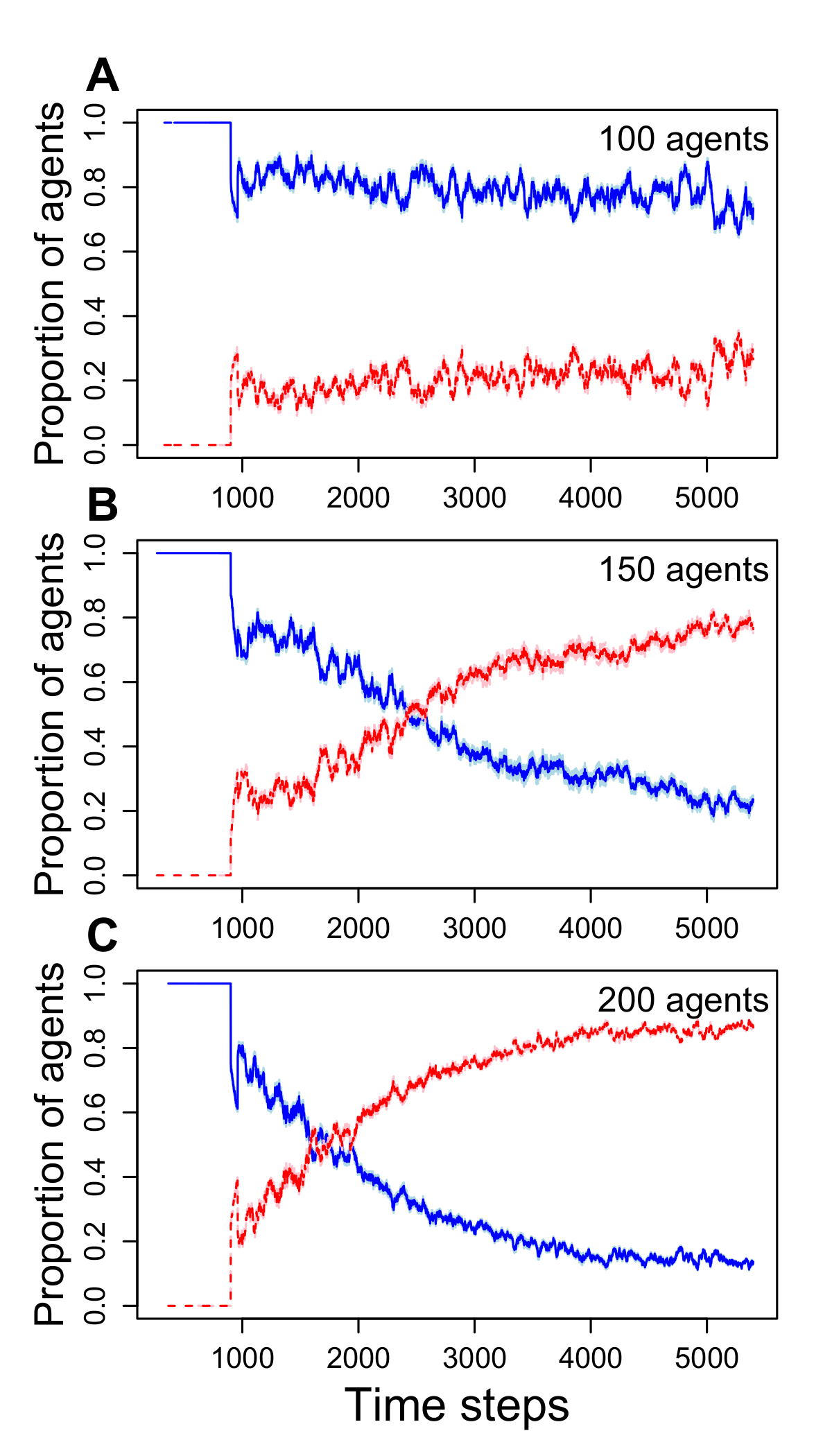

Supplement: Figure S3 — Smallest colony size still showing flexibility under high crowding conditions (8 vs. 24 agents). Proportions of agents foraging at the two food patches, in which the second patch (red line) allowed three times as many agents to feed simultaneously but was made available 900 times steps after agents started foraging at the first food patch (blue line). Data averaged from 10 simulations in each situation. The StDev is shown in light blue and pink. However, since the StDev is very small it is difficult to see by eye. (TIF) [file pone.0044501.s003.tif]

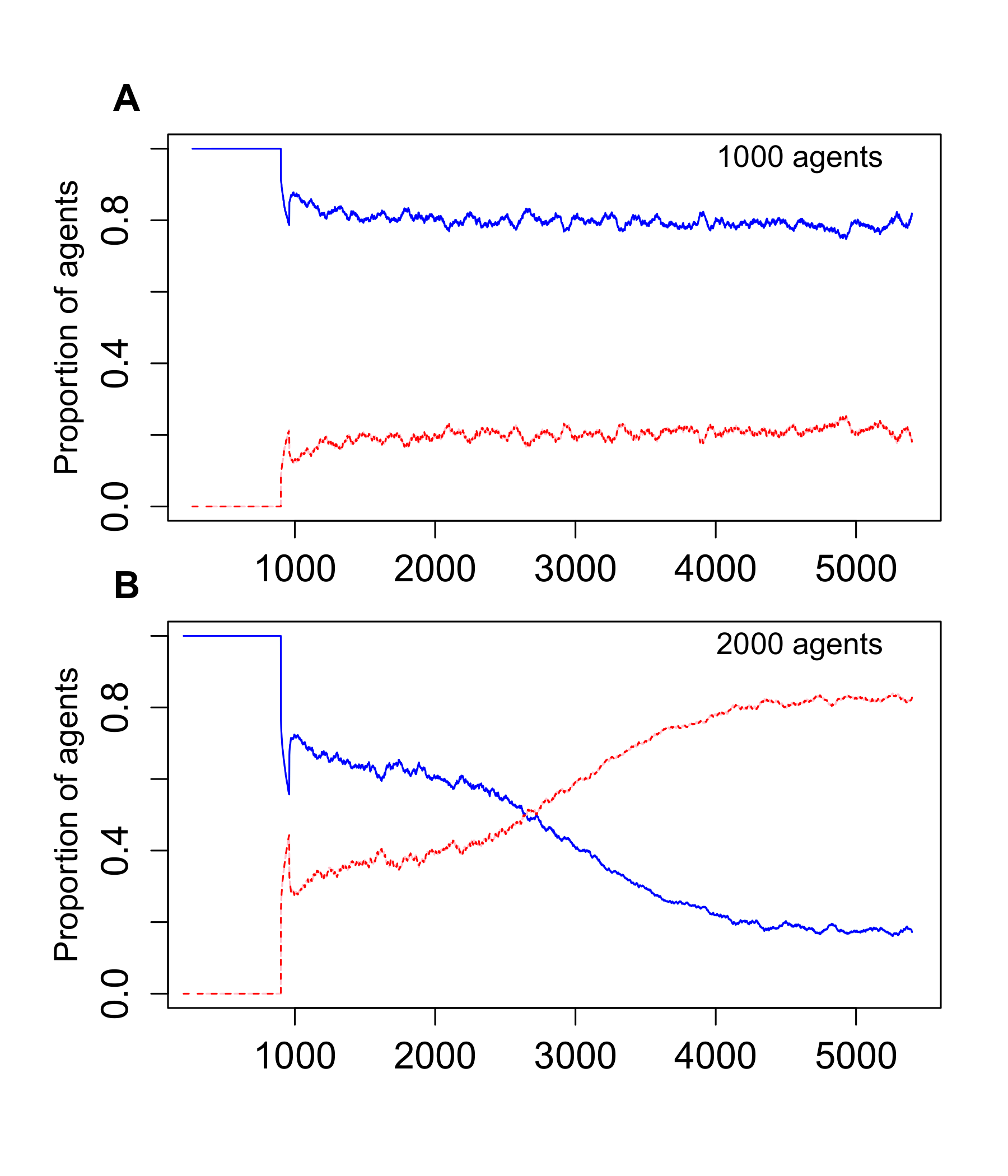

Supplement: Figure S4 — Colony size needed for flexibility under low crowding conditions (72 vs. 216 agents). Proportions of agents foraging at the two food patches, in which the second patch (red line) allowed three times as many agents to feed simultaneously but was made available 900 times steps after agents started foraging at the first food patch (blue line). Data averaged from 10 simulations in each situation. The StDev is shown in light blue and pink. However, since the StDev is very small it is difficult to see by eye. (TIF) [file pone.0044501.s004.tif]

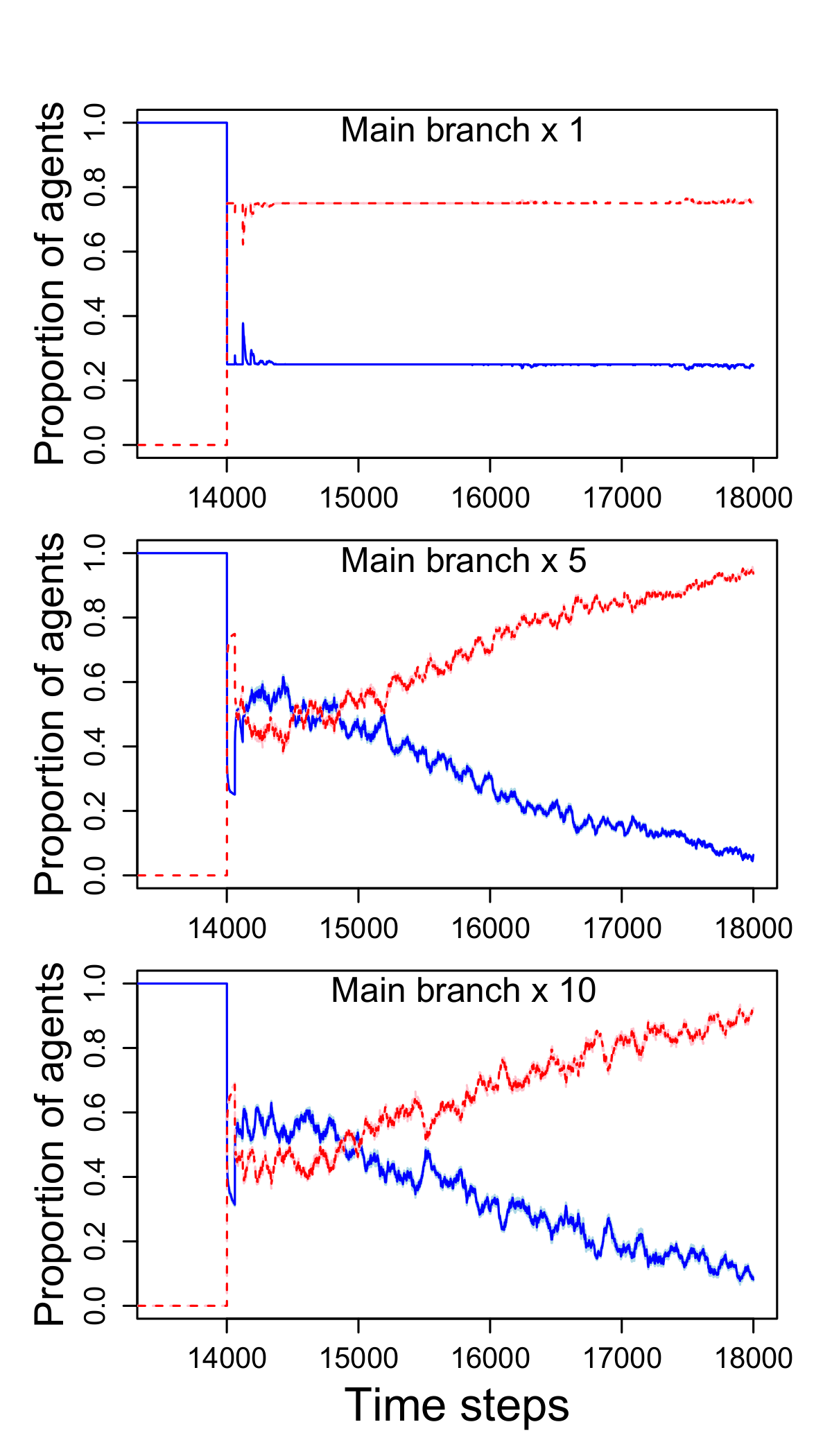

Supplement: Figure S5 — The effect of the main branch length on flexibility under high crowding conditions (8 vs. 24 agents). Proportions of agents foraging at the two food patches, in which the second patch (red line) allowed three times as many agents to feed simultaneously but was made available 14000 time steps after agents started foraging at the first food patch (blue line). The delay of 14000 time steps was chosen because it guaranteed that agents discovered the first food source by random walks even if the main branch was 10 times longer than by default. A main branch length×10 corresponds to approximately 2 m. The instantaneous switch shown in (A) is caused by a large number of dissatisfied agents occupying the second food patch after 14000 time steps. However, with a longer main branch the dissatisfied agents are distributed over a larger area. Data averaged from 10 simulations in each situation. The StDev is shown in light blue and pink. (TIF) [file pone.0044501.s005.tif]

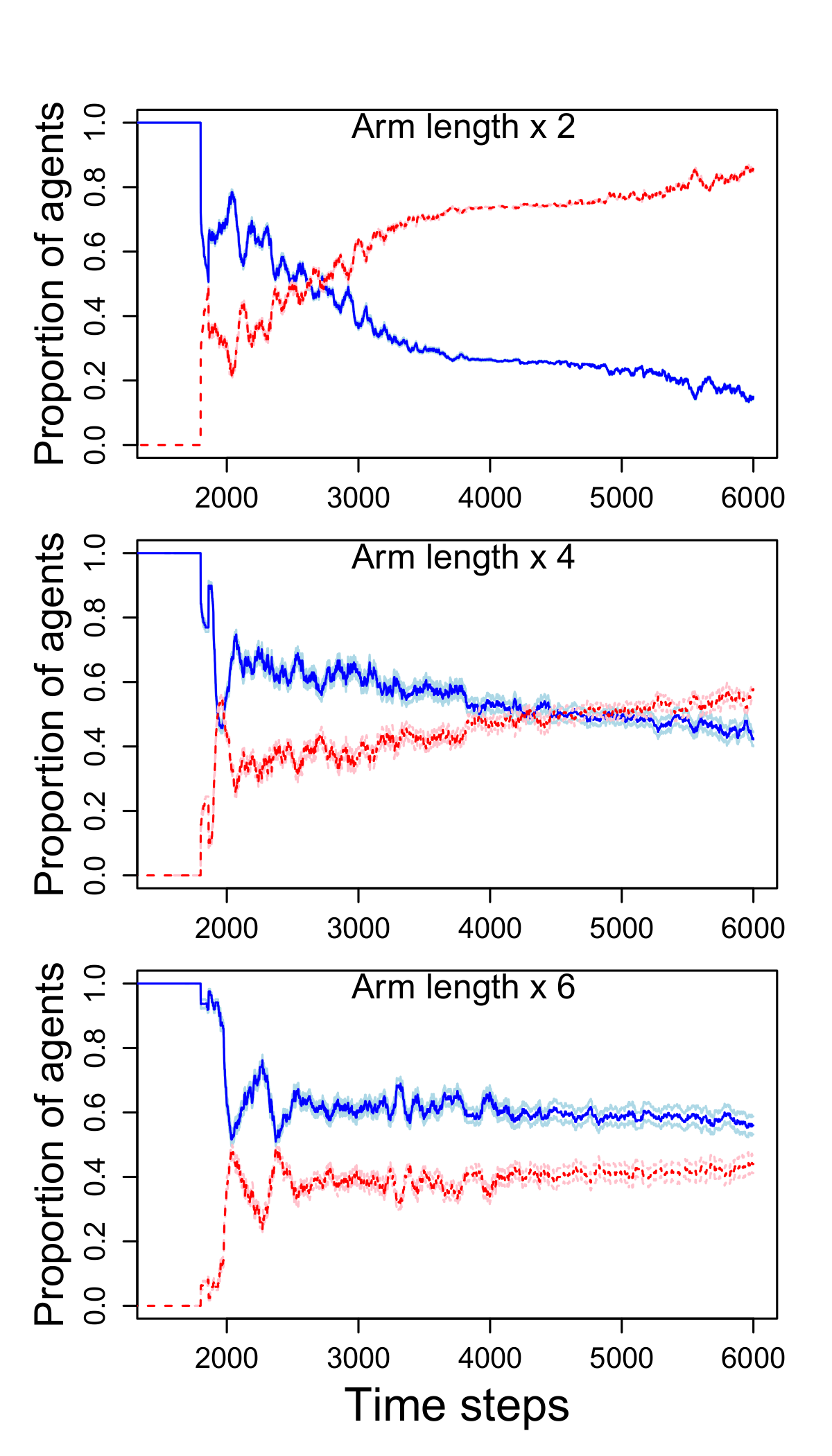

Supplement: Figure S6 — The effect of the arm length on flexibility under high crowding conditions (8 vs. 24 agents). Proportions of agents foraging at the two food patches, in which the second patch (red line) allowed three times as many agents to feed simultaneously but was made available 1800 time steps after agents started foraging at the first food patch (blue line). The delay of 1800 time steps was chosen because it guaranteed that agents discovered the first food source by random walks even if the arm length was 6 times longer than by default. An arm length×6 corresponds to approximately 2 m. Data averaged from 10 simulations in each situation. The StDev is shown in light blue and pink. (TIF) [file pone.0044501.s006.tif]

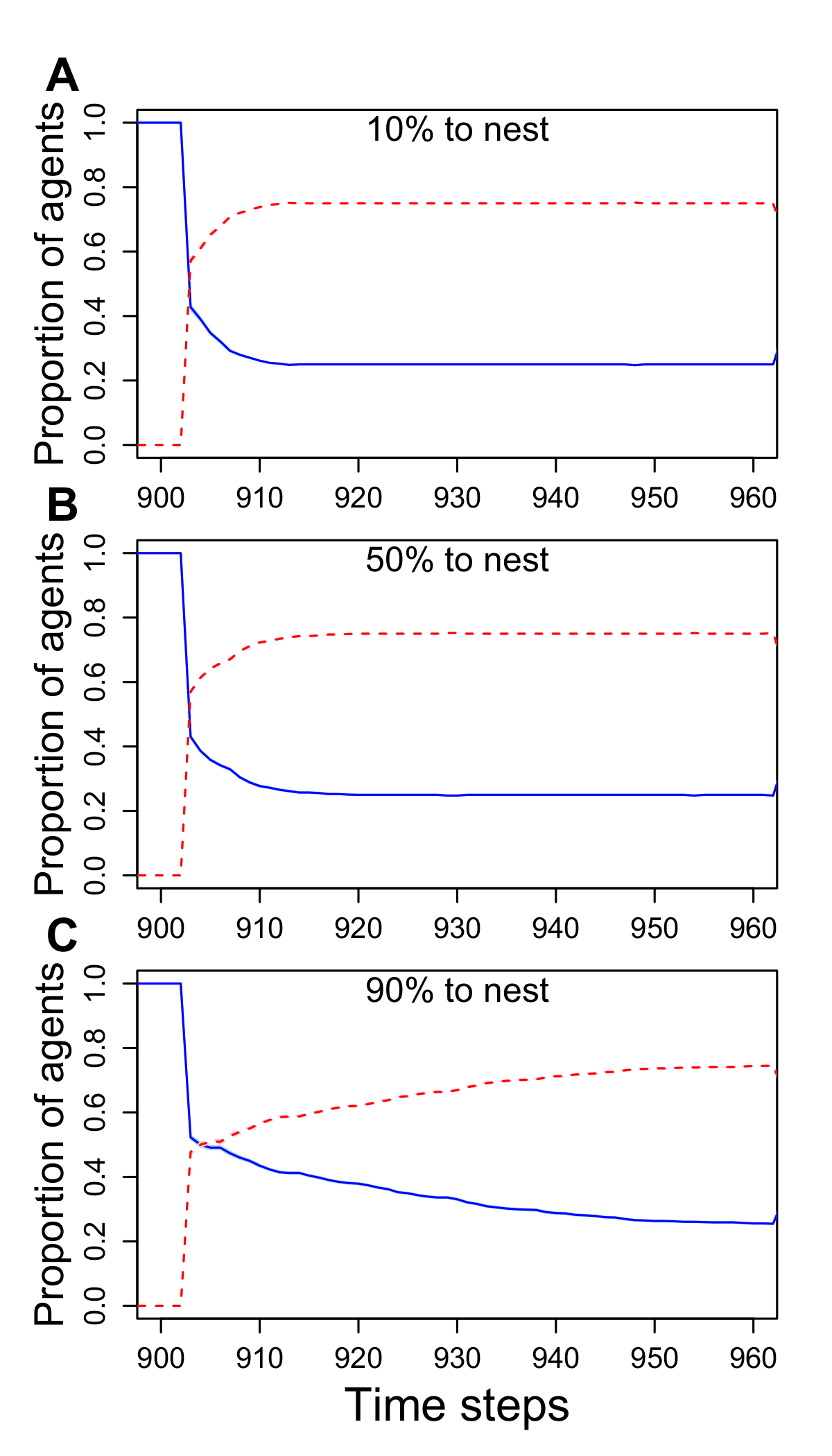

Supplement: Figure S7 — The effect of the probability of dissatisfied agents to walk to the nest versus to the second feeder on collective flexibility under high crowding conditions (8 vs. 24 agents). Proportions of agents foraging at the two food patches, in which the second patch (red line) allowed three times as many agents to feed simultaneously but was made available 900 time steps after agents started foraging at the first food patch (blue line). This model slightly differed from the main model in that dissatisfied ants did not perform a random walk but had a certain probability to either walk on a direct path to the nest or to the second food source (probabilities were 10% vs. 90%, 50% vs. 50, 90% vs. 10%). If both feeders were crowded, dissatisfied agents walked back to the nest and then became “foraging agents” again. Data averaged from 10 simulations in each situation. The StDev is shown in light blue and pink. (TIF) [file pone.0044501.s007.tif]
